# A Peptide Nucleic Acid (PNA) Masking the miR-145-5p Binding Site of the 3′UTR of the Cystic Fibrosis Transmembrane Conductance Regulator (*CFTR*) mRNA Enhances CFTR Expression in Calu-3 Cells

**DOI:** 10.3390/molecules25071677

**Published:** 2020-04-05

**Authors:** Shaiq Sultan, Andrea Rozzi, Jessica Gasparello, Alex Manicardi, Roberto Corradini, Chiara Papi, Alessia Finotti, Ilaria Lampronti, Eva Reali, Giulio Cabrini, Roberto Gambari, Monica Borgatti

**Affiliations:** 1 Department of Life Sciences and Biotechnology, University of Ferrara, 44121 Ferrara, Italy; shaiq.sultan@unife.it (S.S.); jessica.gasparello@unife.it (J.G.); chiara.papi@student.unife.it (C.P.); alessia.finotti@unife.it (A.F.); ilaria.lampronti@unife.it (I.L.); giulio.cabrini@unife.it (G.C.); monica.borgatti@unife.it (M.B.); 2 Department of Chemistry, Life Sciences and Environmental Sustainability, University of Parma, 43124 Parma, Italy; andrea.rozzi@studenti.unipr.it (A.R.); alex.manicardi@unipr.it (A.M.); roberto.corradini@unipr.it (R.C.); 3 IRCCS Istituto Ortopedico Galeazzi, 20161 Milan, Italy; 4 Department of Neurosciences, Biomedicine and Movement, University of Verona, 37134 Verona, Italy; 5 Center of Research on Innovative Therapies for Cystic Fibrosis, University of Ferrara, 44124 Ferrara, Italy

**Keywords:** Peptide nucleic acids, PNA-masking, cystic fibrosis, microRNAs, miR-145-5p, miRNA targeting, delivery, CFTR

## Abstract

Peptide nucleic acids (PNAs) have been demonstrated to be very useful tools for gene regulation at different levels and with different mechanisms of action. In the last few years the use of PNAs for targeting microRNAs (anti-miRNA PNAs) has provided impressive advancements. In particular, targeting of microRNAs involved in the repression of the expression of the cystic fibrosis transmembrane conductance regulator (*CFTR*) gene, which is defective in cystic fibrosis (CF), is a key step in the development of new types of treatment protocols. In addition to the anti-miRNA therapeutic strategy, inhibition of miRNA functions can be reached by masking the miRNA binding sites present within the 3′UTR region of the target mRNAs. The objective of this study was to design a PNA masking the binding site of the microRNA miR-145-5p present within the 3′UTR of the *CFTR* mRNA and to determine its activity in inhibiting miR-145-5p function, with particular focus on the expression of both *CFTR* mRNA and CFTR protein in Calu-3 cells. The results obtained support the concept that the PNA masking the miR-145-5p binding site of the *CFTR* mRNA is able to interfere with miR-145-5p biological functions, leading to both an increase of *CFTR* mRNA and CFTR protein content.

## 1. Introduction

Peptide nucleic acids (PNAs) are DNA analogues of outstanding biological properties [1,2,3,4] since, despite a radical structural change with respect to DNA and RNA, they are capable of sequence-specific and efficient hybridization with complementary nucleic acids, forming Watson–Crick double helices [1]. In addition, they are able to generate triple helices with double stranded DNA and to perform strand invasion [4,5]. Accordingly, they have been used as very efficient tools for pharmacologically-mediated alteration of gene expression, both in vitro and in vivo [6,7,8]. PNA and PNA-based analogues have been proposed as antisense molecules targeting mRNAs, triple-helix forming molecules targeting eukaryotic gene promoters, artificial promoters, and decoy molecules targeting transcription factors [6,7,8,9,10].

Recent published reports strongly support the concept that PNAs can be a very powerful tool to inhibit the expression of microRNAs [11,12,13,14,15,16,17]. MicroRNAs (19 to 25 nucleotides in length) are noncoding RNAs that regulate gene expression by targeting mRNAs, leading to a translational repression or mRNA degradation [18,19,20,21,22,23]. Since their discovery, the number of microRNA sequences present within the miRNA databases has significantly grown [22]. The complex networks constituted by miRNAs and mRNAs lead to the control of highly regulated biological functions, such as differentiation, the cell cycle, and apoptosis [23].

Epigenetic regulation of expression of cystic fibrosis transmembrane conductance regulator (*CFTR*) gene by miRNAs has been recently reported by different groups [24,25,26,27,28,29,30,31,32,33,34]. For instance, expression of miR-145 and miR-494 was found to anti-regulate *CFTR* [28]. The effect of air pollutants and cigarette smoke on *CFTR* expression identified two more miRNAs that could target *CFTR* mRNA, namely miR-101 and miR-144 [25]. Synergistic post-transcriptional regulation of *CFTR* gene expression by miR-101 and miR-494 specific binding was demonstrated [30]. Different miRNAs that have been found to be increased in the primary bronchial epithelial cells of cystic fibrosis (CF) patients can reduce *CFTR* expression, either by direct (miR-145-5p, miR-223-3p, miR-494-3p, miR-509-3p, miR-101-3p) or by indirect (miR-138-5p) interactions. Therefore, targeting miRNAs, such as miR-145-5p, might be an important strategy for upregulating *CFTR*. We have elsewhere published data supporting the use of miR-145-5p targeting in CF, based on an antisense PNA able to enhance expression of the *CFTR* gene, analyzed at the mRNA (RT-qPCR) and protein (Western blotting) levels [33,34]. This conclusion was recently confirmed by Kabir et al., who demonstrated that miR-145 mediates TGF-β inhibition of synthesis and function of the CFTR in CF airway epithelia [35].

In addition to the anti-miRNA therapeutic strategy, an anti-miRNA biological effect can be reached by masking the miRNA binding sites present within the 3′UTR region of the target mRNAs [36,37,38].

The objective of this study was to design a PNA masking the miR-145-5p binding site present within the 3′UTR of the *CFTR* mRNA and to determine its activity in inhibiting miR-145-5p function, with particular focus on the expression of both *CFTR* mRNA and CFTR protein in Calu-3 cells. The PNA was conjugated to a poly-arginine tail, since these types of constructs were previously used by our group for highly efficient delivery of PNA into cell lines [15]. As the experimental model system, the Calu-3 cell line was selected. These cells are a well-differentiated and characterized cell line derived from human bronchial submucosal glands and extensively used to study CFTR expression and immunological behavior [39,40].

## 2. Results

### 2.1. Location of miR-145-5p Binding Sites within the 3′UTR Sequence of CFTR mRNA: Targeting with the miR145-maskingPNA

**Figure 1:**
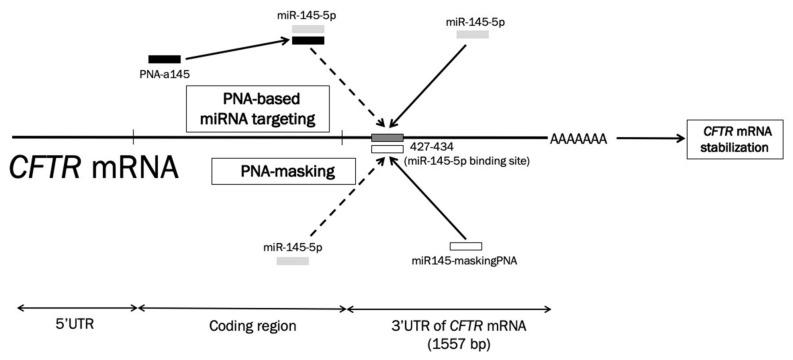
Comparison of the peptide nucleic acid (PNA)-based miRNA-targeting (upper part of the panel) and the PNA-masking (lower part of the panel) strategies to inhibit miR-145-5p biological functions. Dark grey box: the miR-145-5p binding site; light grey boxes: miR-145-5p; white box: miR145-maskingPNA; black boxes: the anti-miR-145-5p PNA-a145. Dotted arrows: inhibition/interference.

Figure 1 shows the location of the miR-145-5p binding site within the 3′UTR sequence (position 427-437 of the 1557 nucleotides long 3′UTR) of the human *CFTR* mRNA [30,31] and the different mechanism of action of PNA-based miRNA targeting (upper part of the panel) versus PNA masking (lower part of the panel). In the case of regulating miR-145-5p by PNA-based miRNA targeting, we elsewhere proposed the use of an anti-miR PNA for targeting miR-145-5p (Figure 1, top). Octaarginine-anti-miR PNA conjugates were delivered to Calu-3 cells, exerting sequence dependent targeting of miR-145-5p. This allowed for the enhanced expression of the miR-145-regulated *CFTR* gene, analyzed at the mRNA (RT-qPCR) and CFTR protein (Western blotting) levels. An alternative strategy for up-regulating CFTR might be the masking of the miR-145-5p binding site with PNAs directed against this sequence (Figure 1, bottom).

### 2.2. Synthesis and Characterization of the miR145-maskingPNA

The synthesis of the miR145-maskingPNA was similar to those previously reported [15,33]. The synthesis was performed using a standard Fmoc-based automatic peptide synthesizer for both the PNA and the polyArg tail. After cleavage from the solid support, purification was performed by HPLC, and the purified PNA was characterized by UPLC/MS. The chemical characterization parameters are reported in the Supplementary Materials (Figure S1). A carrier octaarginine R8 peptide was conjugated at the N-terminus of the PNA chain causing an increase of delivery that approaches 100% (i.e., uptake in 100% of the target cell population), as elsewhere published [15]; this conjugation is easily realized during PNA solid-phase synthesis using the same reagents and solvents.

**Figure 2:**
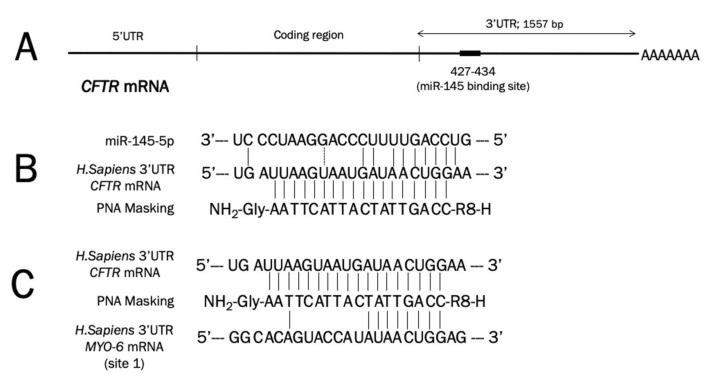
(**A**). Location of the miR-145-5p binding sites within the cystic fibrosis transmembrane conductance regulator (*CFTR*) 3′UTR mRNA region. (**B**). Interactions between the miR145-maskingPNA and *CFTR* mRNA (comparison with the interaction of *CFTR* mRNA with miR-145-5p is also shown). (**C**). Interactions between the miR145-maskingPNA and the *Myosin-6* mRNA [41], containing in the 3′UTR sequence three miR-145-5p binding sites (the miR-145-5p binding site#1 is here shown, which exhibits the highest levels of complementarity to the miR145-maskingPNA). The miR145-maskingPNA is fully complementary with the 3′UTR region of the *CFTR* mRNA.

Figure 2 shows the location of the miR-145-5p binding site (Figure 2A) within the 3′UTR *CFTR* mRNA sequence together with the extent of homology between the miR-145-5p binding site and the miR145-maskingPNA. The design of the miR145-maskingPNA, fully complementary to the miR-145-5p *CFTR* mRNA binding site (Figure 2B), was chosen in order to obtain an efficient competition between miR-145-5p and its 3′UTR *CFTR* mRNA binding sites. In fact, the interaction between this miR145-maskingPNA and the *CFTR* mRNA is expected to be much more efficient than the interaction between the miR-145-5p and *CFTR* mRNA, since the *CFTR* nucleotides complementary to the miR-145-5p are 10/18. On the other hand, the same miR145-maskingPNA exhibits low levels of complementarity to the miR-145-5p binding sites of other mRNAs. For instance, the miR145-maskingPNA exhibits only 9 residues complementary to the 18 nucleotides region of a functional miR-145-5p binding site validated in the 3′UTR region of the *Myosin-6* mRNA [41] (see the bottom part of Figure 2).

### 2.3. Specificity of the miR145-maskingPNA

**Figure 3:**
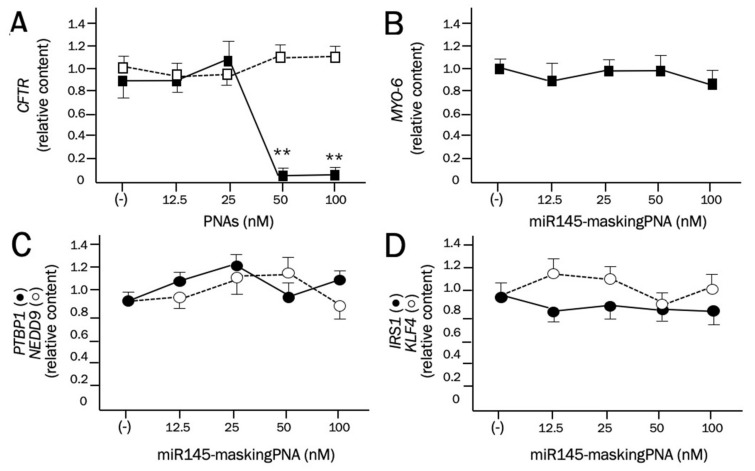
Effects of the miR145-maskingPNA on the RT-PCR amplification of 3′UTR mRNA sequences. (**A**). Effects of the miR145-maskingPNA (black symbols) and of the negative control PNA (white symbols) on the amplification of *CFTR* mRNA sequences. Inhibition by miR145-maskingPNA is clearly seen at 50 nM. The negative control PNA was not effective. Effects of the miR145-maskingPNA on the amplification of *Myosin-6* (**B**), *PTBP1* (polypyrimidine tract binding protein 1) and *NEDD9* (neural precursor cell expressed, developmentally down-regulated 9) (**C**), *IRS1* (insulin receptor substrate 1), and *KLF4* (Kruppel-like factor 4) (**D**) mRNAs. No PCR inhibition in any of these examples was appreciable even at the highest concentrations used.

The specificity of the miR145-maskingPNA is suggested by the experiment depicted in Figure 3. The miR145-maskingPNA was added to cDNAs obtained after RT reactions performed using Calu-3 RNA. The PCR amplification was performed using primers amplifying a 3′UTR region of either *CFTR* (using one primer located on the PNA binding site, Figure 3A) or *Myosin-6* (Figure 3B) mRNAs. As Figure 3 shows, full inhibition of the RT-qPCR amplification of *CFTR* mRNA was obtained when 50 and 100 nM miR145-maskingPNA were employed (Figure 3A). In contrast, no inhibition of amplification was detectable when primers for *Myosin-6* mRNA were used. Furthermore, the miR145-maskingPNA was unable to inhibit the RT-qPCR amplification of other mRNAs carrying miR-145-5p binding sites, including polypyrimidine tract binding protein 1 (*PTBP1*) [42], neural precursor cell expressed, developmentally down-regulated 9 (*NEDD9*) [43], insulin receptor substrate 1 (*IRS1*) [44], and Kruppel-like factor 4 (*KLF4*) [45] mRNAs (Figure 2, C and D). The complementarity of the miR145-maskingPNA with the miR-145-5p binding sites of *PTBP1*, *NEDD9*, *IRS1*, and *KLF4* mRNAs are shown in the Supplementary Materials (Figure S2).

### 2.4. Effects of the miR145-maskingPNA on CFTR Gene Expression

**Figure 4:**
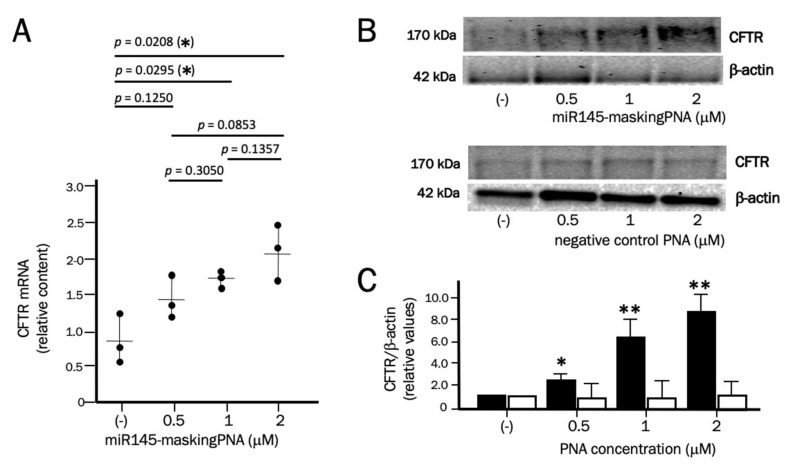
Effects of the miR145-maskingPNA on *CFTR* mRNA (**A**) and CFTR protein (**B**,**C**) in Calu-3 cells. Calu-3 cells were treated with the indicated concentrations of the miR145-maskingPNA for 3 days. Then, RNA was extracted and *CFTR* mRNA content determined by RT-qPCR (**A**). At the same time, CFTR was quantified by Western blotting (**C**, black bars). In parallel, Calu-3 cells were treated with a negative control PNA for 3 days and Western blotting was performed (**C**, white bars). In panel B, representative Western blotting results are shown. In panel C, averages ± SD are shown (*n* = 3) with respect to untreated Calu-3 cells. * = *p* < 0.05; ** = *p* < 0.01 (miR145-maskingPNA vs. negative control PNA).

Calu-3 cells were cultured for 72 h in the presence of different concentrations of the miR145-maskingPNA, then RNA and proteins were isolated for experiments of RT-qPCR and Western blotting. When RT-qPCR was performed, a clear effect was observed on *CFTR* mRNA accumulation. The relative *CFTR* mRNA content in treated Calu-3 cells is reported relative to untreated samples. The relative values of *CFTR* mRNA in untreated samples was calculated with respect to the average *CFTR* mRNA content in control cells. The data obtained show that *CFTR* mRNA increased when Calu-3 cells treated with the miR145-maskingPNA were compared with untreated cells in three independent experiments (Figure 4A). The difference in *CFTR* mRNA content between untreated cells and cells treated with the miR145-maskingPNA is significant (*p* < 0.05 at 1 and 2 μM miR145-maskingPNA). The effects on *CFTR* mRNA were particularly evident, as expected, at the highest concentration of PNA (2.14- to 4.23-fold increase was obtained when 2 μM miR145-maskingPNA were used). Figure 4 (B and C) shows the results of the Western blotting performed using two antibodies, one specific for CFTR, the other for β-actin, used as an internal control. As reported in other published studies [46,47,48,49], the Western blotting analysis based on the CFTR-directed monoclonal antibody 596 shows only a major band corresponding to the fully-glycosylated 170 kDa form of CFTR (known as the C-band) [46,47,48,49].

The CFTR protein increase was found to be 6- to 8-fold in Calu-3 extracts after miR145-maskingPNA treatment in the three independent experiments in which CFTR mRNA was also analyzed (Figure 4B, black boxes). In order to verify specificity of the effects derived by the Western blotting experiment, Calu-3 cells were cultured with a negative control PNA previously demonstrated unable to (a) interact with miR-145-5p [33] and (b) inhibit RT-qPCR amplification of *CFTR* mRNA sequences containing miR-145-5p binding sites (Figure 3, white symbols). The results obtained (Figure 4B) demonstrate no increase of the relative CFTR/β-actin values in Calu-3 cells treated with the negative control PNA. Examples of the raw data used to produce panel C of Figure 4 are shown in the Figure S3 of Supplementary Materials. The data shown in Figure 4C (representing a summary of the different experiments performed) were derived from CFTR/β-actin ratios of treated samples, each expressed relative to the control untreated samples (arbitrarily expressed as 1 in order to compare different independent experiments and different exposures; see Figure S3 for an example of the calculations).

It should be noted that the concentration of the miR145-maskingPNA (Figure 4) was similar to that reported in the case of other anti-miRNA PNAs, but much higher of that used in the arrested-PCR experiments (Figure 3). This is not unexpected when considering the fact that these two strategies are completely different. This difference was also found in one recent paper by our group comparing PNA-based miRNA-arrested PCR with anti-miRNA activity on cultured cell lines [50].

These results suggest that miR-145-maskingPNA should be considered in the development of miRNA-therapeutic protocols for CFTR upregulation.

## 3. Discussion

The data presented in this short report show that a PNA masking the miR-145-5p binding sites present within the 3′UTR of the *CFTR* mRNA is able to increase the expression of the miR-145-5p regulated *CFTR*. The increase of *CFTR* gene expression was detectable at the level of mRNA (analyzed by RT-qPCR) and protein (analyzed by Western blotting). Even if assays on functional activity of the CFTR were not included in the present study, our results could provide a proof-of-principle that miRNA masking might represent an efficient tool to increase CFTR content (possibly by increasing CFTR stability), with possible applications in the personalized therapy of CF. The field of precision medicine is growing. With respect to different molecular and genetic bases of CF, it is expected that miR-145-5p masking will not be useful for CFTR defects of types I (no protein), II (no traffic), or III (no function). In contrast, increase of CFTR levels is expected to be useful for CFTR defects of types IV (less function), V (less protein), and VI (less stable protein). In any case, combined therapy using the miRNA-masking approach with read-through molecules and splicing correctors might be proposed.

For a possible translation to therapeutic approaches for CF, our data are just a proof-of-principle and limited in their application potential. In fact, concerning miRNA masking, we should consider that, in addition to miR-145-5p, several other miRNAs have been proposed to down-regulate CFTR expression, such as miR-494, miR-509-3p, miR-101, and miR-443 [28,30]. Therefore, screening of PNAs targeting the binding sites of these miRNAs, identification of the most active molecules, and combined treatments using the more efficient inhibitor molecules should be considered in order to reach CFTR increases compatible with clinical effects.

As far as the comparison between the miRNA inhibiting and the miRNA masking strategies, we would like to underline that we have elsewhere published data supporting the use of miR-145-5p targeting in CF, based on an antisense PNA to target miR-145-5p and enhance expression of the *CFTR* gene [33,34]. This conclusion was recently confirmed by Kabir et al., who demonstrated that miR-145 mediates TGF-β inhibition of synthesis and function of the CFTR in CF airway epithelia [35]. This direct anti-miRNA strategy is expected to inhibit miR-145-5p function, affecting, in addition to the *CFTR* gene, other miR-145-5p-regulated mRNAs. In contrast, PNA-based miRNA masking might lead to effects restricted to the *CFTR* mRNA and would therefore be of great translational relevance.

Despite the fact that comparison between the anti-miR-PNA and PNA-masking approaches has not been done in parallel in this study, by comparing our results to those published by Fabbri et al. [35], the PNA masking approach appears to be more effective than the anti-miR-PNA approach on the increase of CFTR. The fold increases of CFTR protein were 2- to 2.5-fold and 6- to 8-fold when the anti-miR-PNA [33] and PNA masking (Figure 4) approaches were employed, respectively, when the PNAs were used at 2 μM. Moreover, very low effects using anti-miR-145 PNA were obtained at lower concentrations (unpublished data), while the miR145-maskingPNA was active even when used at 0.5 μM. Comparison with other groups working with anti-miR-145 molecules cannot be performed because these groups employed other anti-miRNA molecules and cell lines [34,35,51].

We underline that this approach should be validated (a) using primary CF-HBE and (b) on *CFTR* mutant cell lines in combination with personalized treatments depending on the CFTR mutations. In case *CFTR* expression can be further increased by treatment with the miR145-maskingPNA, the translational value of the present study will be fully supported for the development of tailored pre-clinical protocols.

## 4. Materials and Methods

### 4.1. Synthesis and Characterization of PNAs

The synthesis and characterization of the miR145-maskingPNA was similar to those previously reported [15] (see Figure S1 of Supplementary materials). The synthesis was performed using a standard Fmoc-based automated peptide synthesizer (Syro I, MultiSynTech GmbH, Witten, Germany), using a ChemMatrix-RinkAmide resin loaded with Fmoc-Gly-OH (0.2 mmol/g) as the first monomer and using commercially available monomers (Link Technologies, Bellshill, UK) with HBTU/DIPEA coupling. Cleavage from the solid support was performed with 10% m-cresol in trifluoroacetic acid, followed by precipitation and washings with diethyl ether. Purification was performed by HPLC using a XTerra Prep RP_18_ (7.8 × 300 mm, 10µm) column. Gradient: 100% A for 5 min, then from 0% to 50% B for 30 min at 4 mL/min flow (A: water + 0.1% trifluoroacetic acid; B: acetonitrile + 0.1% trifluoroacetic acid). After purification, the PNAs were characterized using the following HPLC-MS (Waters, Sesto San Giovanni, Italy) instrumental set-up: Waters Acquity ultra performance LC HO6UPS-823M, with Waters SQ detector and ESI-interface equipped with Waters UPLC BEH 300 (50 × 2.1 mm, 1.7 µm, C18). Chromatographic condition: eluent A: water + 0.2% formic acid; eluent B: CAN + 0.2% formic acid. Column temperature: 35 °C. Program: initial isocratic at 100% A (0.9 min), then linear gradient to 50% B (in 5.7 min). Final wash with 100% B for 1.2 min. Flow rate: 0.25 mL/min. The concentration of the PNA was calculated using UV-absorbance at 260 nm assuming an additive contribution of all bases.

*PNA-1* (*miR145-maskingPNA):* sequence H-R8-CCAGTTATCATTACTTAA-Gly-NH2; yield (after purification): 5% R_t_ = 2.84 min, MS: *calculated MW*: 6135.31; *m/z found (calculated)*: 1228.3 (1228.06) [MH_5_]^5+^, 1023.8 (1023.55) [MH_6_]^6+^, 877.6 (877.47) [MH_7_]^7+^, 768.1 (767.91) [MH_8_]^8+^, 682.8 (682.70) [MH_9_]^9+^, 614.6 (614.53) [MH_10_]^10+^.

### 4.2. Calu-3 Cell Line and Culture Conditions

Calu-3 cells [39,40] (American Type Culture Collection, ATCC HTB-55) were cultured in a humidified atmosphere of 5% CO_2_/air in DMEM/F12 medium (Gibco, Grand Island, NY, USA) supplemented with 10% fetal bovine serum (Biowest, Nauillè, Francia), 100 units/mL penicillin, 100 μg/mL streptomycin (Lonza, Verviers, Belgio), and 1% NEEA (100X) (non-essential amino acids solution; Gibco). To determine the effect on proliferation, cell growth was monitored by determining the cell number/mL using a Z2 Coulter Counter (Coulter Electronics, Hialeah, FL, USA). The sequence of the miR145-maskingPNA is shown in Section 4.1; the sequence of the negative control PNA was H-R8-AGAGATGCCTTGGAGAAC-GLY-NH_2_ (complementarity to the CFTR mRNA and cDNA was lower than 35%).

### 4.3. RNA Extraction

Cultured cells were trypsinized and collected by centrifugation at 1500 rpm for 10 min at 4 °C, washed with PBS, and lysed with Tri-Reagent (Sigma Aldrich, St.Louis, Missouri, USA) according to manufacturer’s instructions. The isolated RNA was washed once with cold 75% ethanol, dried, and dissolved in nuclease free pure water before use.

### 4.4. Arrested PCR for Analysis of the Specificity of the miR145-maskingPNA

Calu-3 cells were used to prepare the cDNA after reverse transcriptase (RT) reactions using the TaqMan MicroRNA reverse transcription kit (Applied Biosystem). This cDNA was incubated with miR145-maskingPNA in respective quantities of 12.5 nM, 25 nM, 50 nM, and 100 nM for 2–3 min; following a real time PCR reaction with 3′UTR specific primers of *CFTR* mRNA (F-5′-TGC AAG CCA GAT TTT CC-3′, R-5′-GTT TCC AGT TAT CAT TAC TTA A-3′), *MYO-6* mRNA (F-5′-AGG AAG AAA CAA AAC AGT G-3′, R-5′-CTG ATT TTC CAC TTA AGA TG-3′), *NEDD9* mRNA (F-5′-TTG GCC CAG TTC TTA TTT AGC -3′, R-5′- TGG CAG AGT AGG ACT TTG AG-3′), *IRS1* mRNA (F-5′-ATG AGA GCA GAA ATG AAC AGA C-3′, R-5′-TGA GTA CCA GCA ACT TCC AG-3′), *PTBP1* mRNA (F-5′-TAA TCA AGT CAC GTG ATT-3′, R-5′- AGT TAC TTA AAA CTA TTT CT-3′), and *KLF4* mRNA (F-5′-AAT GGT TTA TTC CCA AG-3′, R-5′-ACT TAA TTC TCA CCT TGA -3′) (Integrated DNA Technologies, Coralville, USA). To identify the 3′UTR regions of target mRNAs, the UTRdb site was used [41]. All reactions, including no-PNA controls and RT-minus controls were performed in duplicates using the CFX-96 Touch Real-time detection system (Bio-Rad, Hercules, CA, USA). The relative expression was calculated using the comparative cycle threshold method.

### 4.5. Analysis of CFTR Expression: RT-qPCR

Gene expression analysis was performed by RT-qPCR. First, 300 ng of the total RNA was reverse transcribed by using random hexamers. Quantitative real-time PCR (qPCR) assays were carried out using gene-specific double fluorescently labeled probes. Primers and probes used to assay *CFTR* (Assay ID: Hs00357011_m1) gene expression were purchased from Applied Biosystems, (Applied Biosystems, Foster City, CA, USA). The relative expression was calculated using the comparative cycle threshold method and, as reference genes, the human *RPL13A* (Assay ID: Hs03043885_g1) [33].

### 4.6. Analysis of CFTR Expression: Western Blotting

Cell pellets were lysed in a 1% Nonidet P40 (IGEPAL), 0.5% sodiumdeoxycholate, 200mM NaCl, 10mM Trizma base, pH 7.8, and 1 mM EDTA plus protease inhibitor mixture, and then sonicated with 1mM PMSF for 30 min in ice. Lysates were cleared by centrifugation at 10,000× *g* for 10 min at 4 °C. Protein concentration was determined by the BCA method after precipitation with 5% trichloroacetic acid (TCA), utilizing bovine serum albumin as a standard. For CFTR analysis, 40 μg of total protein was heated in XT sample buffer 4x (Bio-Rad Laboratories, Hercules, CA, USA) at 37 °C for 10 min and loaded onto a 3% to 8% tris-acetate gel (Bio-Rad Laboratories, Hercules, CA, USA). The gel proteins were transferred to PVDF membrane (Bio-Rad Laboratories, Hercules, CA, USA) by using a Trans Blot Turbo (Bio-Rad Laboratories, Hercules, CA, USA). In our protocol, the gels were cut in two pieces, one containing materials with molecular weights higher than 75 kDa (for CFTR analysis), the other containing proteins between 37 kDa and 75 kDa (for β-actin analysis). For CFTR analysis, the Western blotting filter was processed using the mouse monoclonal antibody, clone 596, against the NBD2 domain of CFTR (University of North Carolina, Cystic Fibrosis Center, Chapel Hill, NC, US) at a dilution of 1:2500 by an overnight incubation at 4 °C. After washes, the membranes were incubated with horseradish peroxidase-coupled anti-mouse immunoglobulin (R&D System, Minneapolis, MN, USA) at room temperature for 1 h and after subsequent washes, the signal was developed by enhanced chemiluminescence (LumiGlo Reagent and Peroxide, Cell Signaling). For β-actin analysis, the 37–70 kDa filter was processed with an anti β-actin monoclonal antibody (rabbit mAb-13E5, Cell Signaling Technology, Leiden, The Netherlands) in order to confirm the equal loading of samples. This antibody was used at a dilution of 1:1000 by an overnight incubation at 4 °C and, after washes, the membranes were incubated with horseradish peroxidase-coupled anti-rabbit immunoglobulin (Cell Signaling Technology, Leiden, The Netherlands).

### 4.7. Statistical Analysis

Results are expressed as average ± standard deviation (S.D.). Comparisons between groups were made by using paired Student’s *t* test. Statistical significance was defined with *p* < 0.05 (*, significant) and *p* < 0.01 (**; highly significant).

## Supplementary Materials

The following are available online at https://www.mdpi.com/1420-3049/25/7/1677/s1.

## Abbreviations

CFTR: cystic fibrosis transmembrane conductance regulator; miRNA: microRNA; PNA: peptide nucleic acid; RT-PCR: reverse transcription polymerase-chain reaction; SDS: sodium dodecylsulphate; SDS-PAGE: SDS-polyacrylamide-gel electrophoresis.
