# Supplementary material for: A Peptide Nucleic Acid (PNA) Masking the miR-145-5p Binding Site of the 3′UTR of the Cystic Fibrosis Transmembrane Conductance Regulator (CFTR) mRNA Enhances CFTR Expression in Calu-3 Cells
Source: Molecules. 2020 Apr 5;25(7):1677. doi: 10.3390/molecules25071677 (PMC7181265; doi:10.3390/molecules25071677)
Supplement: Supplementary file 1 [file molecules-25-01677-s001.pdf]

A Peptide nucleic acid (PNA) masking the miR-145-5p binding site of the 3'-UTR of the Cystic Fibrosis Transmembrane Conductance Regulator (CFTR) mRNA enhances CFTR expression in Calu-3 cells

Shaiq Sultan[1], Andrea Rozzi[2], Jessica Gasparello[1], Alex Manicardi[2,3], Roberto Corradini[2], Chiara Papi[1], Alessia Finotti[1], Ilaria Lampronti[1], Eva Reali[4], Giulio Cabrini[1,5], Roberto Gambari[1,6,*] and Monica Borgatti[1]

[1] Department of Life Sciences and Biotechnology, University of Ferrara, 44121, Ferrara, Italy;

[2] Department of Chemistry, Life Sciences and Environmental Sustainability, University of Parma, 43124, Parma, Italy;

[3] Present address: Department of Organic and Macromolecular Chemistry, University of Ghent, 9000, Gent, Belgium;

[4] IRCCS Istituto Ortopedico Galeazzi, 20161 Milan, Italy;

[5] Department of Neurosciences, Biomedicine and Movement, University of Verona, 37134, Verona, Italy;

[6] Center of Research on Innovative Therapies for Cystic Fibrosis, University of Ferrara, 44124, Ferrara, Italy;

Correspondence to:

Professor Roberto Gambari, Department of Life Sciences and Biotechnology, University of Ferrara, Via Fossato di Mortara n.74, 44121 Ferrara, Italy; Tel: +39-532-974443; Fax: +39-532-974500; email: gam@unife.it.

Supplementary Material

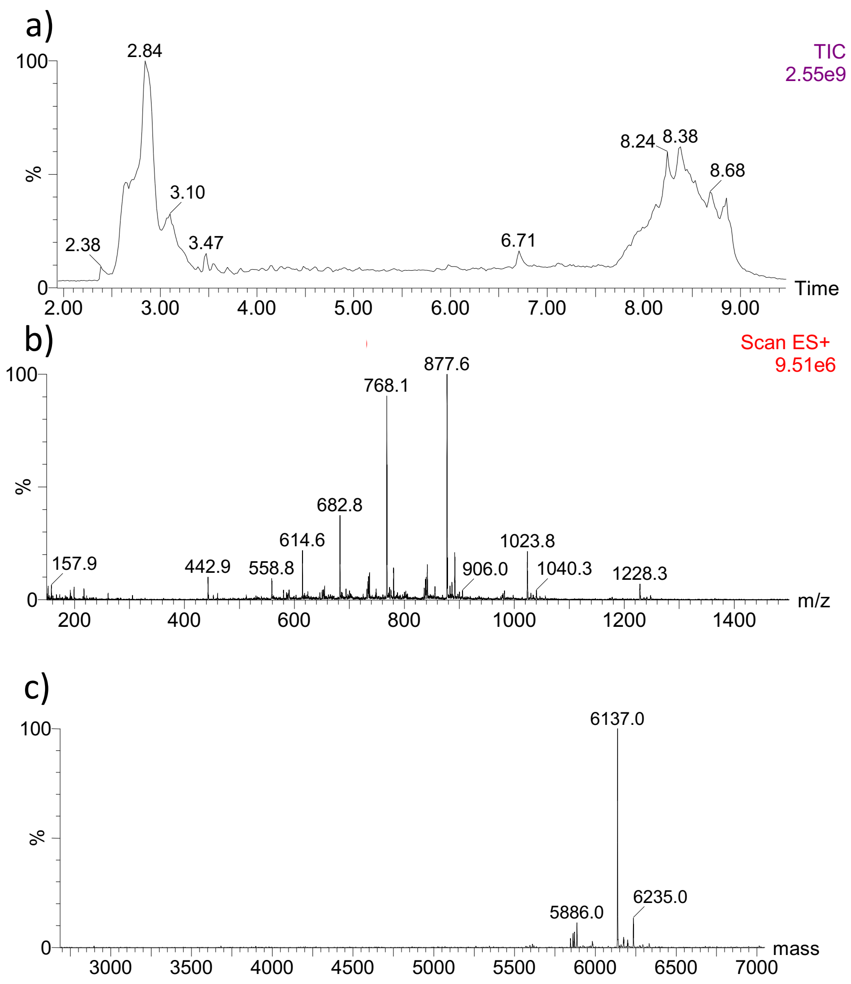

Figure S1. (a) UPLC/MS Chromatogram of the purified ***PNA-1*** (“***miR145-maskingPNA”)***, used for the present work; peak at 2.84 min corresponds to the target PNA. (b) Mass spectrum of the peak at 2.84 min; reconstructed molecular mass obtained from deconvolution of the spectrum in (c).

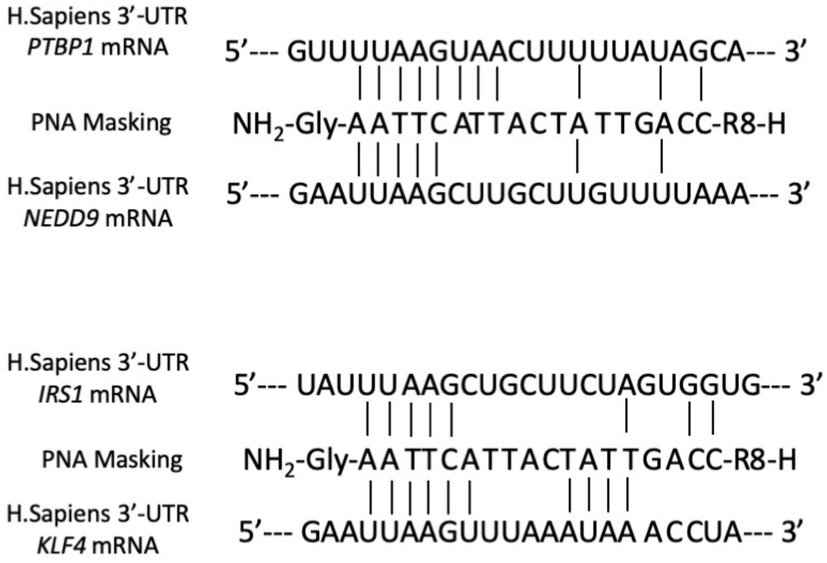

Figure S2. Interactions between the miR145-maskingPNA and the PTBP1 (Polypyrimidine Tract Binding Protein 1) [1], NEDD9 (Neural Precursor Cell Expressed, Developmentally Down-Regulated 9) [2], IRS1 (Insulin Receptor Substrate 1) [3] and KLF4 (Kruppel Like Factor 4) [4] mRNAs. No inhibition in all these examples is appreciable even at the highest concentrations used (Figure 3 of the main text). The miR145-maskingPNA is fully complementary with the 3’UTR region of the CFTR mRNA (Figure 2 of the main text).

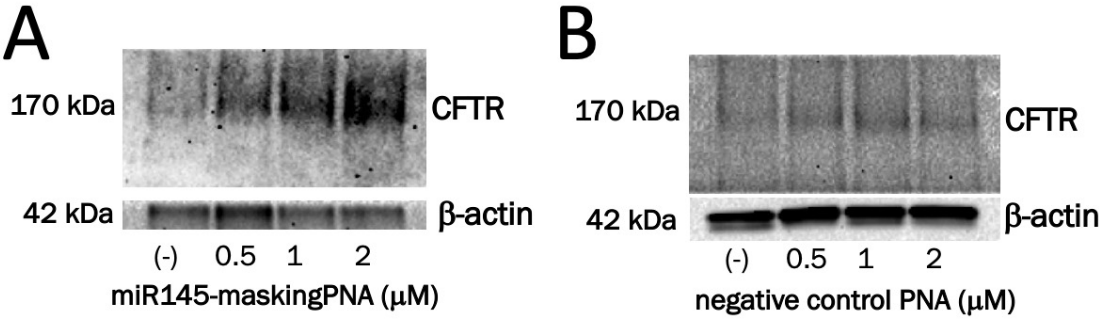

C

miR145-masking PNA (μM)	CFTR		β-actin		CFTR/β-actin	
	Adjusted volume	Relative value	Adjusted volume	Relative value	Absolute value	Relative value
(-)	126,108	1.00	97,196	1.00	1.30	1.00
0.5	436,248	3.46	177,952	1.83	2.45	1.88
1	695,628	5.52	107,875	1.11	6.45	4.96
2	981,528	7.78	108,031	1.11	9.09	6.99

D

negative control PNA (μM)	CFTR		β-actin		CFTR/β-actin	
	Adjusted volume	Relative value	Adjusted volume	Relative value	Absolute value	Relative value
(-)	174,678	1.00	104,604	1.00	1.67	1.00
0.5	164,166	0.94	117,353	1.12	1.40	0.84
1	171,925	0.98	105,605	1.01	1.63	0.98
2	178,731	1.02	103,619	0.99	1.72	1.03

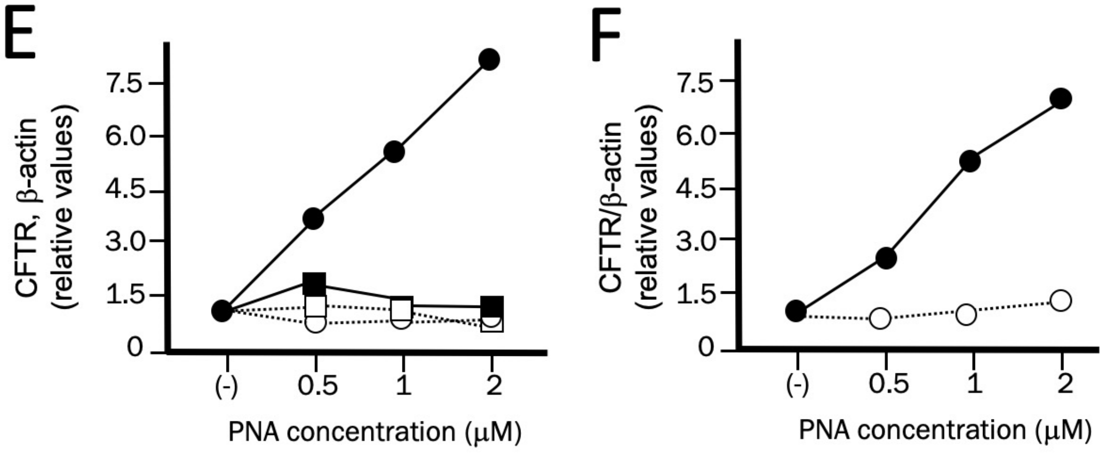

Figure S3. **Increase of CFTR expression in Calu-3 cells treated with the miR145-maskingPNA.** Raw data of the Western blotting performed using the CFTR- and β-actin directed monoclonal antibodies are presented in panels A-D. Calu-3 cells have been treated for 3 days with the indicated concentrations of miR145-maskingPNA and negative control PNA, as indicated. Panels A and B show the original autoradiography film obtained after exposure of the filter, treated with the CFTR- and β-actin specific antibody, to horseradish peroxidase (HRP) substrate; panel C and D show raw data and representative calculations relative to the densitometric analysis of the autoradiography films shown in A and B. E,F. Relative values of Adjusted volumes (E) and of the calculated CFTR/β-actin ratios (F). Treatment with the miR145-maskingPNA: black symbols; treatment with the negative control PNA: open symbols; CFTR, circles; β-actin: squares.

Results

Densitomeric analysis of Western blotting. For densitometry analysis, the ChemiDoc XRS+ System (Bio-Rad) was used. The Image Lab Software for ChemiDoc and Gel Doc Imaging Systems (Version 6.0.1) was employed in order to acquire, display, edit and analyze the Western blot images, and to display and export data [5,6]. In our case, we performed all the analyses without ambiguity relative to the obtained results. In order to calculate the correct volumes, tools were used to highlight the band section and subtract the background signal. Further, the whole volume analysis Table was saved in excel file to perform further statistical analysis. The raw data of the Western blot experiment shown in Figure 4 (B and C) are reported in the upper part of Figure S3. An increase of the Adjusted volume values was found only when samples from Calu-3 cells treated with miR145-maskingPNA were bound to the CFTR-directed monoclonal antibody 596. No increase in the Adjusted volume values was found in samples from cells cultured in the presence of the negative control PNA. On the other hand, the Adjusted volume values relative to β-actin did not exhibit major changes in samples isolated from cells treated with the two PNAs. In the last two columns the CFTR/β-actin ratios (including both absolute and relative values) are shown. These calculations generated the summary panel depicted in Figure 4C.

References

[1] Minami K, Taniguchi K, Sugito N, Kuranaga Y, Inamoto T, Takahara K, Takai T, Yoshikawa Y, Kiyama S, Akao Y, Azuma H. MiR-145 negatively regulates Warburg effect by silencing KLF4 and PTBP1 in bladder cancer cells. Oncotarget 2017;8:33064-33077.

[2] Speranza MC, Frattini V, Pisati F, Kapetis D, Porrati P, Eoli M, Pellegatta S, Finocchiaro G. NEDD9, a novel target of miR-145, increases the invasiveness of glioblastoma. Oncotarget 2012;3:723-34.

[3] Wang Y, Hu C, Cheng J, Chen B, Ke Q, Lv Z, Wu J, Zhou Y. MicroRNA-145 suppresses hepatocellular carcinoma by targeting IRS1 and its downstream Akt signaling. Biochem Biophys Res Commun. 2014;446:1255-60.

[4] Liu H, Lin H, Zhang L, Sun Q, Yuan G, Zhang L, Chen S, Chen Z. miR-145 and miR-143 regulate odontoblast differentiation through targeting Klf4 and Osx genes in a feedback loop. J Biol Chem. 2013;288:9261-71.

[5] Taylor SC, Berkelman T, Yadav G, Hammond M. A defined methodology for reliable quantification of Western blot data. Mol Biotechnol. 2013;55:217-26.

[6] Beekman C, Janson A, Baghat A, van Deutekom J, Datson N. Use of capillary Western immunoassay (Wes) for quantification of dystrophin levels in skeletal muscle of healthy controls and individuals with Becker and Duchenne muscular dystrophy. PLoS ONE. 2018;13:e0195850.
